# A pilot randomised controlled trial of personalised care for depressed patients with symptomatic coronary heart disease in South London general practices: the UPBEAT-UK RCT protocol and recruitment

**DOI:** 10.1186/1471-244X-12-58

**Published:** 2012-06-06

**Authors:** André Tylee, Mark Haddad, Elizabeth Barley, Mark Ashworth, June Brown, John Chambers, Anne Farmer, Zoe Fortune, Rebecca Lawton, Morven Leese, Anthony Mann, Paul McCrone, Joanna Murray, Carmine Pariante, Rachel Phillips, Diana Rose, Gill Rowlands, Ramon Sabes-Figuera, Alison Smith, Paul Walters

**Affiliations:** 1 Health Services and Population Research Dept, Institute of Psychiatry at King’s College London, De Crespigny Park, London, SE5 8AF, UK; 2 Department of Primary Care and Public Health Sciences, King’s College London, 9th Floor, Capital House, 42 Weston Street, London, SE1 3QD, UK; 3 Department of Psychology, Institute of Psychiatry at King’s College London, De Crespigny Park, London, SE5 8AF, UK; 4 Department of Cardiology, Guy’s and St Thomas’ Hospitals, Westminster Bridge Road, London, SE17EH, UK; 5 Department of Social Genetic and Developmental Psychiatry, Institute of Psychiatry at King’s College London, De Crespigny Park, London, SE5 8AF, UK; 6 Department of Psychological Medicine, Institute of Psychiatry at King’s College London, De Crespigny Park, London, SE5 8AF, UK; 7 Faculty of Health and Social Care, London South Bank University, 103 Borough Road, London, SE1 0AA, UK

## Abstract

**Background:**

Community studies reveal people with coronary heart disease (CHD) are twice as likely to be depressed as the general population and that this co-morbidity negatively affects the course and outcome of both conditions. There is evidence for the efficacy of collaborative care and case management for depression treatment, and whilst NICE guidelines recommend these approaches only where depression has not responded to psychological, pharmacological, or combined treatments, these care approaches may be particularly relevant to the needs of people with CHD and depression in the earlier stages of stepped care in primary care settings.

**Methods:**

This pilot randomised controlled trial will evaluate whether a simple intervention involving a personalised care plan, elements of case management and regular telephone review is a feasible and acceptable intervention that leads to better mental and physical health outcomes for these patients. The comparator group will be usual general practitioner (GP) care.

81 participants have been recruited from CHD registers of 15 South London general practices. Eligible participants have probable major depression identified by a score of ≥8 on the Hospital Anxiety and Depression Scale depression subscale (HADS-D) together with symptomatic CHD identified using the Modified Rose Angina Questionnaire.

Consenting participants are randomly allocated to usual care or the personalised care intervention which involves a comprehensive assessment of each participant’s physical and mental health needs which are documented in a care plan, followed by regular telephone reviews by the case manager over a 6-month period. At each review, the intervention participant’s mood, function and identified problems are reviewed and the case manager uses evidence based behaviour change techniques to facilitate achievement of goals specified by the patient with the aim of increasing the patient’s self efficacy to solve their problems.

Depressive symptoms measured by HADS score will be collected at baseline and 1, 6- and 12 months post randomisation. Other outcomes include CHD symptoms, quality of life, wellbeing and health service utilisation.

**Discussion:**

This practical and patient-focused intervention is potentially an effective and accessible approach to the health and social care needs of people with depression and CHD in primary care.

**Trial registration:**

ISRCTN21615909.

## Background

Coronary heart disease (CHD) is a common chronic disease, affecting around 3.5% of the UK population [1]; it is the most common cause of mortality in the world [2] and in the UK accounted for 18% of premature deaths in men and 9% in women in 2008 [3]. It was ranked as the second leading cause of disability in high- and middle-income countries in 2004 [2].

Depression is likewise an important public health problem: it has a 12-month prevalence of around 4% [4] and is currently the leading cause of global disease burden in high- and middle-income countries [2]. For at least half of all people who experience an episode, depression is characterised by relapses, and for 10 - 20% it involves chronic symptoms [5,6]. Depression co-occurs in a substantial proportion of patients with chronic medical conditions including CHD; and this co-occurrence is associated with poorer quality of life and increased morbidity and mortality [7,8]. There is consistent evidence that depression is predictive of subsequent coronary events in people with established CHD and the risk of fatal cardiac events in this population is more than doubled when this is co-morbid with depression [7]. Mortality risk appears most strongly associated with depression onset following the acute coronary syndrome [9].

The association between depression and CHD appears to be related to physiological mechanisms such as altered inflammatory responses and changes in platelet aggregation [10], as well as to a range of health behaviours. Factors such as sedentary lifestyle, unhealthy diet, cigarette smoking and reduced adherence to exercise or medication regimens elevate the risk of adverse health outcomes [11]. Providing interventions that facilitate changes in health behaviour as well as impacting on depression has clear relevance for this patient group.

In the UK, most people with depression are treated in primary care [12], however there is limited provision within current services for its longer-term management, or for care that addresses its frequent co-morbidity with medical conditions. Improving the management of depression in people with CHD is an important goal, with the potential for benefit for both psychological and physical health outcomes. Researchers have investigated the effectiveness of pharmacological and psychological therapies with patients experiencing this combination of conditions, and systematic reviews conducted for the recent National Institute for Health and Clinical Excellence clinical guideline clearly show that these treatment approaches are effective on depression outcomes in comparison with standard care [11]. There is also emerging evidence for the benefit of these types of intervention on a range of physical health outcomes including glycaemic control [13], low-density lipoprotein cholesterol, and systolic blood pressure [14].

There is particular value in a coordinated treatment approach that addresses the physical and psychological needs of this patient group within primary care, and avoids the fragmentation of care delivery that can hamper the management of long-term conditions [15]. The links between depression and CHD are likely to be complex, but lifestyle factors appear especially important for cardiac outcomes as well as for quality of life [16]. It appears that certain interventions used in chronic disease management are of particular relevance. Components such as education about the condition, interventions to encourage physical exercise, systematic monitoring of the patient’s response and concordance to treatment, and assisting lifestyle modification have been found to be associated with psychological improvement as well as benefiting physical health. A recent systematic review has identified benefit for depression outcomes from such community-based heart disease interventions [17].

The current study evaluates a novel personalised care approach using regular pro-active contact and follow-up and involving elements of *case management*. The term ‘case management’ was first used in the 1950s in the USA to describe the extended community services needed for discharged mental health patients, and has subsequently developed to become a widely-used approach for managing the care required by people with complex health and social care needs [18]. It is a systematic proactive approach used to assess and organize care using a health professional (typically a nurse or social worker), the case manager, to work collaboratively with the patient to plan and monitor treatments and supports. Key elements of this role involve using scheduled follow-ups to help develop self-management techniques; providing education about the illnesses; supporting ongoing treatments whilst addressing non-adherence or lack of improvement by adjustments or facilitating changes; and where appropriate coordinating care across the health and social care system [19]. In England, NHS case management has been a central aspect of the long-term conditions strategy, and is focussed explicitly on the care of those people with complex long-term needs who are at high risk of decline and unplanned reactive specialist care such as hospital admissions [20].

Within the current study, the personalised care approach is more broadly focused than case management which typically exclusively targets the most complex and vulnerable patients; and is delivered by means of regular telephone contacts. It is centred on collaborative working underpinned by a care plan agreed between the patient and case manager and incorporates appropriate and accessible health education and the support for self-care, using evidence based behaviour change techniques that are noted in UK health policy as the main approach to care for the majority of people with long-term conditions. The intention within this study is to evaluate an intervention that could be feasibly delivered by health professionals in primary care such as practice nurses who are in an excellent position to provide such an approach to patients in the management of chronic problems [21]. Discussion of cases between the case manager and an academic GP will be used each week to mirror the type of case review that is available within the primary care team.

The intervention is designed to enhance current practice with only minimal additional administration and training. It was developed on the basis of reviews of the relevant literature [22] and qualitative studies of patients’ and health professionals’ views on treatment needs for CHD and comorbid depression. This study forms part of a broader programme of research including a prospective cohort study of 803 primary care patients with coronary heart disease to explore the relationship with depression and a nested case–control study to investigate genetic and blood-biomarkers as predictors of depression [23].

## Aims

This study seeks to examine the feasibility and acceptability of telephone-delivered personalised care compared with treatment as usual (TAU) for a subset of primary care CHD patients who have probable concurrent depression, and to explore the types of needs and problems identified by patients in collaboration with their case manager. The methods of the trial will also be tested to inform a definitive trial. The primary outcome to be tested will be the severity of depression features measured by the Hospital Anxiety and Depression Scale[24] depression sub-scale (HADS-D) at 1-, 6-, and 12-months following randomisation. Secondary outcomes tested will include measures of CHD symptoms, wellbeing, and health service utilisation.

## Methods/design

This study is a randomised controlled trial with randomisation by individual patients within practices recruited by the Primary Care Research Network-Greater London. The comparison is between usual primary care (control arm), and personalised care involving regular telephone follow-up by nursing health professionals in addition to usual primary care (intervention arm).

## Sample size

Although estimation of a definitive effect size is not the focus of this pilot study, a sample size calculation indicated that to show a mean difference between intervention and control of ≥ 3 on the HADS Depression subscale, assuming a pooled standard deviation around mean scores of 4, 30 participants per group are needed for 90% power at the 5% significance level. This is also in line with the minimum of 12 per group suggested for pilot studies [25]. To allow for loss to follow-up estimated at 25%, our plan was to recruit 80 participants (40 per arm) into the RCT.

## Recruitment - practices

Practices in South London were eligible for inclusion if they kept a register of patients with CHD for the Quality and Outcomes Framework (QOF) and were willing to be involved in liaison concerning patients in the intervention arm where necessary. Practices already involved in the UPBEAT-UK cohort study were excluded. Recruitment commenced in October 2010. 15 general practices were approached by the Greater London Primary Care Research Network (PCRN-GL), one of eight local primary care research networks for England funded by the National Institute for Health Research Clinical Research Network (NIHR CRN) which assist in coordinating and supporting NHS primary care research, and all practices agreed to participate.

## Recruitment - participants

**Figure 1:**
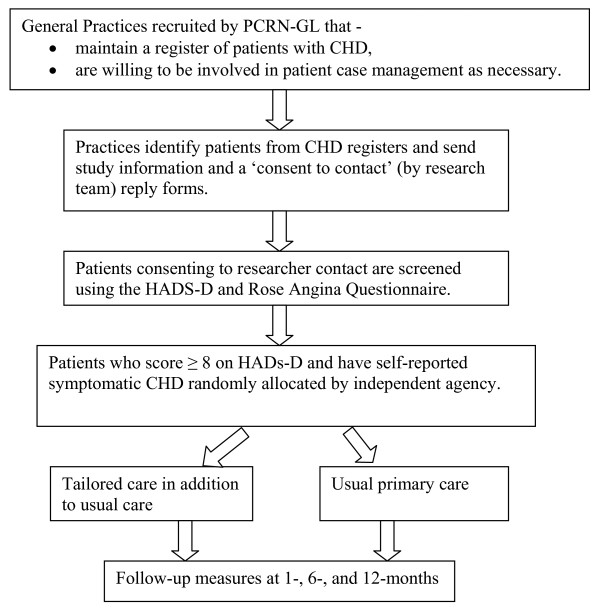
Flow chart of the UPBEAT-UK trial.

All patients on practice case registers for CHD were sent study information and consent to contact requests by participating general practitioners. Those patients who provided consent were contacted by study researchers and assessed for depression using the HADS-D and for symptoms relating to CHD using the Modified Rose Angina Questionnaire [26]. Patients with symptomatic CHD (i.e. current chest pain) and a score of ≥8 on the HADS-D scale [24]were eligible to participate in the study. Those providing fully informed consent to participate were then randomly allocated to either to the intervention (personalised care) or the treatment as usual (TAU) arm of the study (Figure 1).

· Symptomatic CHD as scored on the modified Rose Angina Questionnaire [26]

· A score ≥8 on the depression part of the Hospital Anxiety and Depression Scale [24]

· Aged 18 years or over

· Temporary registrations

· Actively suicidal patients

· Psychotic depression as evidenced by delusions and/or hallucinations

· Non-English speaking

· Participants currently in hospital for treatment of their CHD

## Randomisation

Randomisation was conducted independently by the Mental Health and Neurosciences Clinical Trials Unit (CTU) at King's College London and allocation concealment was ensured by the CTU independent database programme. Randomisation was at patient level. A random permuted block design was used to approximately balance the numbers in the control and intervention groups.

## Intervention

The intervention was developed following a literature review and qualitative studies that explored the views of patients and service providers concerning health needs and care delivery suitable for coronary heart disease and co-morbid depression. Patients’ perspectives on treatments were examined by semi-structured interview with 30 participants of the UPBEAT-UK cohort study with probable depression (identified by PHQ-9 score) in addition to CHD, and by two focus groups involving participants with symptomatic CHD (identified by Rose Angina Questionnaire) and probable depression (by PHQ-9 score). Interviews were conducted with 12 practice nurses and 10 GPs working in practices in 4 ethnically and culturally diverse parts of South London. These studies indicated that an individualised case management-based intervention of a type that could be delivered by practice nurses was likely to be acceptable and effective.

On the basis of these investigations, we developed a tailored face-to-face assessment and telephone-delivered follow-up provided by clinically qualified case managers. Because this was an exploratory study, the case managers were members of the research team, and rather than practice nurses they were a community psychiatric nurse and a health psychologist who is also a registered nurse. Neither of the case managers is involved in collecting the follow-up data.

The case managers arrange to meet with each participant randomised to the intervention arm for an initial assessment. Central to the care model is developing a shared plan of care, and in this study the initial assessment meeting between the patient and case manager is focused on developing a personalised care plan (PCP) based on a comprehensive assessment (see Additional file 1). The format of the assessment materials has been specifically based on the outcome areas identified in the Department of Health green paper *Independence, Well-being and Choice*[27], enabling a multi-factorial assessment of each patient’s physical, mental and social wellbeing and relevant environmental factors. This assessment follows UK developments towards a common assessment framework for adults [28]. A copy of the PCP is kept by the participant, a copy by the case manager and a copy is sent to the participant’s GP. The case manager helps the participant choose up to two main problems on which to work. The focus will be on helping the patients to identify problems they think are contributing to their depression, rather than the case manager imposing their views. The case manager will provide health advice, but a key aim is to develop the patient’s confidence in identifying and solving their own problems. The case managers will be familiar with a range of evidence based behaviour change interventions such as goal setting and rating importance and confidence to change (importance/confidence ruler), which they will use to enhance the patient’s self- efficacy in self management. The case managers also provide written information where necessary about depression, CHD and other health problems, and about appropriate local resources for participants to access, as well as liaising with other health professionals involved in the participant’s care as appropriate.

The initial assessment will last up to an hour, and patients will be followed up by the case manager by telephone (or email, if the patient prefers) for 6 months. Contacts are planned to be at weekly or two-weekly intervals, although the frequency will be reviewed in relation to the patient’s needs and actual intervals will be recorded. During the follow-up, the participant’s PCP will be reviewed with particular emphasis on mutually prioritised problems and new goals will be set in collaboration with the participant as appropriate; each telephone follow-up session is planned to last between 10 and 30 min, but actual durations will be recorded.

## Comparator

Participants randomised to the control group will receive treatment as usual by their GP and any other relevant professionals including the full range of physical and mental healthcare services that may be available in their locality.

## Measures

Participants will be followed up at 1-, 6- and 12-months post randomisation.

The primary outcome measure is depression status and severity identified by the depression subscale items of the Hospital Anxiety and Depression Scale (HADS) [24]. This rating scale is comprised of seven items and has been widely used to examine depression in community populations and particularly among people with co-existing medical conditions.

Additionally a number of secondary outcome measures will be used to examine the efficacy of this intervention and the appropriateness and acceptability of measurement instruments (Table 1). These will include measures of CHD symptoms, well-being and quality of life, and use of health services. These variables will all be measured at baseline, and the three follow-up time-points.

**Table 1:** Measures used at each time point in the UPBEAT-UK randomised controlled trial

**Outcome Parameter**	**Instruments**
**Primary Outcome**	
Depression	HADS-depression subscale [24]
**Secondary Outcomes**	
Depression	PHQ-9 [29]
Coronary Heart Disease	Modified Rose Angina Questionnaire [26], Specific Activity Schedule [30], Guy’s Hospital Chest Pain Questionnaire [31]
Quality of Life	Euroqol 5day [32], Medical Outcomes Survey Short Form-12 (SF-12) [33]
Adherence to medication	Adapted version of Morisky adherence questionnaire [34]
Life events	List of Threatening Events Questionnaire [35]
Social problems	Social Problems Questionnaire [36]
Health Service Utilisation	Client Service Receipt Inventory (CSRI) [37]
Illness Perceptions	Brief Illness Perceptions Questionnaire [38]
Participants’ problem priorities	Psychlops [39]
Wellbeing	Warwick-Edinburgh Mental Well-being Scale [40]

## Blinding

In a randomised comparison of this type, patient blinding is clearly impossible. The researchers will however be blind to randomisation status, and will be asked to give their opinion on randomisation status to determine whether blinding is adequate. If researcher unblinding occurs, a pair of patients (including the patient whose allocation status may have been identified) will be switched to another researcher within the team.

## Statistical analysis

Descriptive analyses will be used to provide summary estimates of outcome measures, focussing on the dropout rate at each time point. A linear mixed effect model for longitudinal data will be used to estimate (using maximum likelihood) between treatment arms the difference in HADS-D scores at 1, 6 and 12 months overall (taking account of any time trends). Intention to treat analysis will be used. While the sample size will not be sufficient to test clustering effects formally, sources of clustering (such as the patient’s GP practice) and approximate size of inter-cluster correlation coefficient will be identified so that they can be taken into account in any future definitive trial.

## Results of recruitment

**Figure 2:**
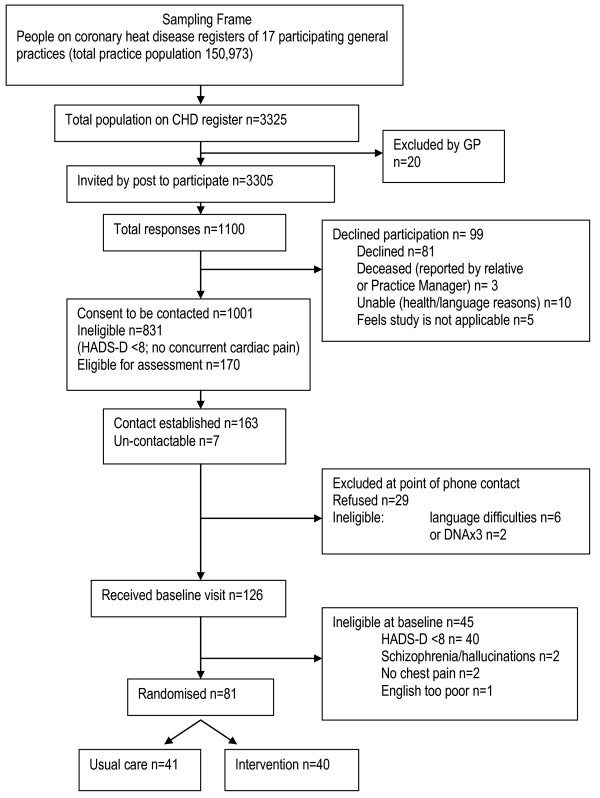
Consort diagram: recruitment and treatment group allocation.

Recruitment ceased in May 2011. The recruitment target was achieved, with 81 eligible patients from 15 practices consenting to participation. Figure 2 details recruitment at each stage, reasons for exclusion and numbers randomised.

## Ethical approval

This trial received ethical approval from the South East London Research Ethics Committee (REC Ref No. 10/H0808/51).

## Discussion

This pilot RCT recruited 81 participants within the South London area over an 8-month period. The recruitment method using structured searches of general practice QOF databases for potential participants who were then approached by means of a letter sent by the practice resulted in the planned number of patients being recruited. This method of recruitment therefore appears to be feasible and effective. The ease of recruitment of both practices and patients suggests that the intervention is perceived as acceptable and potentially useful by both clinicians and depressed people with CHD (Fig. 2).

The intervention has been designed on the basis of a qualitative examination of patient and provider views and reviews of approaches to managing depression and long-term conditions in primary care. It aims to address health and social care needs in a practical, achievable and patient-focused way. The numbers recruited should allow us to examine the feasibility and acceptability of this approach to care, to ascertain the size of effects associated with this intervention and to determine the optimum design of a definitive trial. Direct contact with patients not opting in may have improved recruitment even further as it is possible that depressed patients may have not replied even if they had been potentially interested in the intervention.

## Competing interests

The authors declare they have no competing interests

## Authors’ contributions

All authors participated in the conception and design of this pilot RCT. All authors read and approved the final manuscript.

## Pre-publication history

The pre-publication history for this paper can be accessed here:

http://www.biomedcentral.com/1471-244X/12/58/prepub

## Supplementary Material

Additional file 1 UPBEAT-UK: Personalised Health Plan for Heart and Mind). Click here for file

## References

[B1] Quality and Outcomes Framework Achievement Data 2008/09 2009 The Health and Social Care Information Centre, http://www.ic.nhs.uk/webfiles/QOF/2008-09/QOF%20Achievement%20and%20Prevalence%20Bulletin%202008-09.pdf

[B2] The global burden of disease: 2004 update 2008 WHO, Geneva

[B3] Scarborough P Bhatnagar P Wickramasinghe K Smolina K Mitchell C Rayner M Coronary heart disease statistics 2010 edition 2010 British Heart Foundation, London http://www.heartstats.org/datapage.asp?id=9075

[B4] Waraich P Goldner EM Somers JM Hsu L Prevalence and incidence studies of mood disorders: a systematic review of the literature Can J Psychiatry 2004 49 124 138 15065747 10.1177/070674370404900208

[B5] Hebergen D Batelaan NM de Graaf R Nolen WA Spijker J Beekman AT The 7-year course of depression and anxiety in the general population Acta Psychiatr Scand 2011 123 4 297 306 10.1111/j.1600-0447.2011.01677.x 21294714

[B6] Judd LL Akiskal HS Maser JD Zeller PJ Endicott J Coryell W A prospective 12-year study of subsyndromal and syndromal depressive symptoms in unipolar major depressive disorders Arch Gen Psychiatry 1998 55 694 700 10.1001/archpsyc.55.8.694 9707379

[B7] Barth J Schumacher M Herrmann-Lingen C Depression as a risk factor for mortality in patients with coronary heart disease: a meta-analysis Psychosom Med 2004 66 802 813 10.1097/01.psy.0000146332.53619.b2 15564343

[B8] Stafford L Berk M Reddy P Jackson HJ Comorbid depression and health-related quality of life in patients with coronary artery disease J Psychosom Res 2007 62 401 410 10.1016/j.jpsychores.2006.12.009 17383491

[B9] Poole L Dickens C Steptoe A The puzzle of depression and acute coronary syndrome: reviewing the role of acute inflammation J Psychosom Res 2011 71 61 68 10.1016/j.jpsychores.2010.12.009 21767684 PMC3143279

[B10] Pariante CM Lightman SL The HPA axis in major depression: classical theories and new developments Trends Neurosci 2008 31 464 468 10.1016/j.tins.2008.06.006 18675469

[B11] Depression in adults with a chronic physical health problem: treatment and management (National Clinical Practice Guideline 91) 2009 National Institute for Health and Clinical Excellence, London

[B12] Haddad M Walters P Tylee A Mood disorders in primary care Psychiatry 2009 8 71 75 10.1016/j.mppsy.2008.11.001

[B13] van der Feltz-Cornelis CM Nuyen J Stoop C Chan J Jacobson AM Katon W Effect of interventions for major depressive disorder and significant depressive symptoms in patients with diabetes mellitus: a systematic review and meta-analysis Gen Hosp Psychiatry 2010 32 380 395 10.1016/j.genhosppsych.2010.03.011 20633742

[B14] Katon WJ Lin EH Von Michael K Ciechanowski P Ludman EJ Young B Collaborative care for patients with depression and chronic illnesses N Engl J Med 2010 363 2611 2620 10.1056/NEJMoa1003955 21190455 PMC3312811

[B15] Tylee A Haddad M Managing complex problems: treatment for common mental disorders in the UK Epidemiol Psichiatr Soc 2007 16 302 308 18333426 10.1017/s1121189x00002487

[B16] Frasure-Smith N Lesperance F Depression and cardiac risk: present status and future directions Postgrad Med J 2010 86 193 196 20354040 10.1136/hrt.2009.186957

[B17] Kang-Yi CD Gellis ZD A systematic review of community-based health interventions on depression for older adults with heart disease Aging Ment Health 2010 14 1 19 10.1080/13607860903421003 20155517

[B18] Evans C Drennan V Roberts J Practice nurses and older people: a case management approach to care J Adv Nurs 2005 51 343 352 10.1111/j.1365-2648.2005.03504.x 16086803

[B19] Reilly S Hughes J Challis D Case management for long-term conditions: implementation and processes Ageing & Soc 2010 30 125 155 10.1017/S0144686X09990183 20426316

[B20] Improving Chronic Disease Management 2004 Department of Health, London

[B21] Buszewicz M Griffin M McMahon EM Beecham J King M Evaluation of a system of structured, pro-active care for chronic depression in primary care: a randomised controlled trial BMC Psychiatry 2010 10 61 10.1186/1471-244X-10-61 20684786 PMC2923105

[B22] Barley EA Murray J Walters P Tylee A Managing depression in primary care: A meta-synthesis of qualitative and quantitative research from the UK to identify barriers and facilitators BMC Fam Pract 2011 12 47 10.1186/1471-2296-12-47 21658214 PMC3135545

[B23] Tylee A Ashworth M Barley E Brown J Chambers J Farmer A Up-Beat UK: A programme of research into the relationship between coronary heart disease and depression in primary care patients BMC Fam Pract 2011 12 38 10.1186/1471-2296-12-38 21605435 PMC3120657

[B24] Zigmond AS Snaith RP The hospital anxiety and depression scale Acta Psychiatr Scand 1983 67 361 370 10.1111/j.1600-0447.1983.tb09716.x 6880820

[B25] Tennant Julious SA: Sample size of 12 per group rule of thumb for a pilot study Pharmaceut Statist 2005 4 287 291 10.1002/pst.185

[B26] Rose GA The diagnosis of ischaemic heart pain and intermittent claudication in field surveys Bull World Health Organ 1962 27 645 658 13974778 PMC2555832

[B27] Independence, Well-being and Choice: Our Vision for the Future of Social Care for Adults in England 2005 Department of Health, London

[B28] Common Assessment Framework for Adults: a consultation on proposals to improve information sharing around multi-disciplinary assessment and care planning 2009 Department of Health, London

[B29] Kroenke K Spitzer RL Williams JB The PHQ-9: validity of a brief depression severity measure J Gen Intern Med 2001 16 606 613 10.1046/j.1525-1497.2001.016009606.x 11556941 PMC1495268

[B30] Goldman L Hashimoto B Cook EF Loscalzo A Comparative reproducibility and validity of systems for assessing cardiovascular functional class: advantages of a new specific activity scale Circulation 1981 64 1227 1234 10.1161/01.CIR.64.6.1227 7296795

[B31] Cooke RA Smeeton N Chambers JB Comparative study of chest pain characteristics in patients with normal and abnormal coronary angiograms Heart 1997 78 142 146 9326987 10.1136/hrt.78.2.142 PMC484893

[B32] Rabin R de CF EQ-5D: a measure of health status from the EuroQol Group Ann Med 2001 33 337 343 10.3109/07853890109002087 11491192

[B33] Ware J Kosinski M Keller SD A 12-Item Short-Form Health Survey: construction of scales and preliminary tests of reliability and validity Med Care 1996 34 220 233 10.1097/00005650-199603000-00003 8628042

[B34] Morisky DE Green LW Levine DM Concurrent and predictive validity of a self-reported measure of medication adherence Med Care 1986 24 67 74 10.1097/00005650-198601000-00007 3945130

[B35] Brugha TS Cragg D The List of Threatening Experiences: the reliability and validity of a brief life events questionnaire Acta Psychiatr Scand 1990 82 77 81 10.1111/j.1600-0447.1990.tb01360.x 2399824

[B36] Corney RH Clare AW The construction, development and testing of a self-report questionnaire to identify social problems Psychol Med 1985 15 637 649 10.1017/S0033291700031494 4048322

[B37] Beecham J Knapp M Costing psychiatric interventions 2001 Gaskell, In Measuring Mental Health Needs. Edited by Thornicroft G. London

[B38] Broadbent E Petrie KJ Main J Weinman J The brief illness perception questionnaire J Psychosom Res 2006 60 631 637 10.1016/j.jpsychores.2005.10.020 16731240

[B39] Ashworth M Shepherd M Christey J Matthews V Wright K Parmentier H A client-generated psychometric instrument: The development of 'PSYCHLOPS' Couns Psychother Res 2004 4 27 31 10.1080/14733140412331383913

[B40] Tennant R Hiller L Fishwick R Platt S Joseph S Weich S The Warwick-Edinburgh Mental Well-being Scale (WEMWBS): development and UK validation Health Qual Life Outcomes 2007 5 63 10.1186/1477-7525-5-63 18042300 PMC2222612

