# Supplementary material for: A pilot randomised controlled trial of personalised care for depressed patients with symptomatic coronary heart disease in South London general practices: the UPBEAT-UK RCT protocol and recruitment
Source: BMC Psychiatry. 2012 Jun 6;12:58. doi: 10.1186/1471-244X-12-58 (PMC3437191; doi:10.1186/1471-244X-12-58)
Supplement: Additional file 1 — UPBEAT-UK: Personalised Health Plan for Heart and Mind). [file 1471-244X-12-58-S1.doc]

| **UPBEAT-UK: Personalised Health Plan for Heart and Mind** | | |
| --- | --- | --- |
| **Date of assessment**  **Identifying details** | | |
| | Name |  |  | Date of birth |  | | --- | --- | --- | --- | --- | |  |  |  |  |  | | Male | Female |  | Contact tel. no. |  | |  |  |  |  |  | | Address |  |  | Marital status |  | |  |  |  | |  | Dependents |  | |  |  |  | |  | | NHS Number | | --- | |  | |  |  |  | | Postcode |  |  |  |  | | | |
| | Mobility restrictions |  |  | | --- | --- | --- | |  |  |  | | | |
| | Employment status |  | | --- | --- | | | |
| Assessment information | | |
| | Place of assessment (GP Practice/home).  People present at assessment. |  | | --- | --- | | | |
| **Details of person(s) undertaking assessment** | | |
| | Name |  |  | Contact tel. no. |  | | --- | --- | --- | --- | --- | | | |
|  | | |
| | Address |  |  | Role |  | | --- | --- | --- | --- | --- | |  | Organisation |  | | | |
|  | | |
| Postcode: |  |  |
|  | | |
| **Services working with this person** | | |
| | **Universal** | GP/usual GP |  | Details |  | Tel | |  | | --- | | | --- | --- | --- | --- | --- | --- | --- | --- | | Other primary care professionals/usual contact |  | Details |  | Tel | |  | | --- | | | **Other services** | Service |  | Details |  | Tel | |  | | --- | | | Service |  | Details |  | Tel | |  | | --- | | | | |

| | **Your health and how you manage it** | | | --- | --- | | | **Disabilities or conditions (e.g. heart disease)** | | | --- | --- | | **1a: Physical health** | | | **impact on independence or well-being** |  | |  | | General physical well‑being |  | | Sensory problems (e.g. hearing, eye sight) |  | | Medication problems or side effects, including sexual side effects |  | | Sexual problems |  | | Breathing |  | | Sleeping |  | | Pain |  | | Communication needs |  | | | **1b: Psychological and emotional health** | **impact on independence or well-being** | | --- | --- | | | | Mental health needs |  | | Mood |  | | Alcohol, smoking, other substances |  | | | **2: Daily activities** | **impact on independence or well-being** | | --- | --- | | | | Dressing/undressing; Personal hygiene |  | | Doing housework/ daily tasks |  | | Eating and drinking |  | | Mobility indoors;  mobility outside |  | | | **3: Choice and control** | **impact on independence or well-being** | | --- | --- | | | | Care preferences |  | | | **4: Threat or exclusion** | **impact on independence or well-being** | | --- | --- | | | | Security or risks or threats |  | | | **5a:**  **Housing situation** | **impact on independence or well-being** | | --- | --- | | | | Support, costs |  | | Independence, suitability |  | | | **5b: Keeping healthy** | **impact on independence or well-being** | | --- | --- | | | | Exercise |  | | Diet |  | | Carer relationships |  | | | **6a: Education, training, employment** | **impact on independence or well-being** | | --- | --- | | | | Employment/ voluntary involvements |  | | Education courses/ support needs |  | | | **6b: Social involvements** | **impact on independence or well-being** | | --- | --- | | | | Family/friends |  | | Social, cultural, religious involvements |  | | | **7: Finances and Benefits** | **impact on independence or well-being** | | --- | --- | | | | Problems, anticipated changes |  | | Assistance/ support if in hospital |  | |
| --- | --- | --- | --- | --- | --- | --- | --- | --- | --- | --- | --- | --- | --- | --- | --- | --- | --- | --- | --- | --- | --- | --- | --- | --- | --- | --- | --- | --- | --- | --- | --- | --- | --- | --- | --- | --- | --- | --- | --- | --- | --- | --- | --- | --- | --- | --- | --- | --- | --- | --- | --- | --- | --- | --- | --- | --- | --- | --- | --- | --- | --- | --- | --- | --- | --- | --- | --- | --- | --- | --- | --- | --- | --- | --- | --- | --- | --- | --- | --- | --- | --- | --- | --- | --- | --- | --- | --- | --- | --- | --- | --- | --- | --- | --- | --- | --- | --- | --- | --- | --- | --- | --- |

Personal Health Plan Date………….

|  | Problem | Services, professionals, resources involved | **Action**  **(by whom)** | **Review**  **date** |
| --- | --- | --- | --- | --- |
| **Physical health** |  |  |  |  |
| **Mental health** |  |  |  |  |
| **Daily activities** |  |  |  |  |
| **Care preferences** | n/a |  |  |  |
| **Problems accessing or making use of services** |  |  |  |  |
| **Housing situation & quality of life** |  |  |  |  |
| **Education, training, employment & social involvements** |  |  |  |  |
| **Finances & benefits** |  |  |  |  |

| **My Health Plan Goals**   | **Myself:** |  | | --- | --- |  | **My case manager** |  | | --- | --- |  | **Their contact details:** |  | | --- | --- |  | **Plan start *(date)*:** |  | | --- | --- |  | **The issues I would like to address are *(one or two issues based on discussion):*** |  | | --- | --- |   **Specific targets to achieve**   | **1. Target –what you would like to achieve (based on identified problems)** |  | | --- | --- |  | **Plan to achieve this target:**  Try to clearly note the action - with detail of when and how, and who/what will help |  | | --- | --- |  | **2. Target –what you would like to achieve** |  | | --- | --- |  | **Plan to achieve this target:**  Try to clearly note the action - with detail of when and how, and who/what will help |  | | --- | --- |   Review 1   | I will review these targets/goals with my case manager on (date): |  | | --- | --- |  | s/he will ring me on this number |  | | --- | --- |   Review 2   | I will review these targets/goals with my case manager on (date): | Weekly/ 2- weekly. | | --- | --- |  | s/he will ring me on this number |  | | --- | --- |   Review 3   | I will review these targets/goals with my case manager on (date): |  | | --- | --- |  | s/he will ring me on this number |  | | --- | --- |   **Successes and Difficulties**  Before speaking to your case manager, please record your thoughts about achieving your goals:   | Please write here anything you have done in relation to your goals that you are pleased about. |  | | --- | --- |  | Please write here any difficulties that you have had working on your goals. | | |  | | | | | | | | | | | |  | | --- | --- | --- | --- | --- | --- | --- | --- | --- | --- | --- | --- | --- | --- | --- | --- | |  | **Consent statement for information storage and information sharing**  “We will treat your information as confidential and we will not share it with any other organisation unless we are required by law to share it or unless you will come to some harm if we do not share it. In any case we will only ever share the minimum information we need to share”  I understand the information that is recorded on this form and that it will be stored and used for the purpose of providing services for me | | | | | | | | | | | | |  | | |  | I agree to the sharing of information, as agreed, between the services listed below | | | | | | | Yes | | |  | No |  |  | | |  | |  | | --- | | | | | | | | | | | | | | | | |  | Signed |  | | Name |  | | Date | | |  | | | |  | | |  | **Case Manager’s signature** | | | | | | | | | | | | |  | | |  | Signed |  | | Name |  | Date | | |  | | | | |  | | |
| --- | --- | --- | --- | --- | --- | --- | --- | --- | --- | --- | --- | --- | --- | --- | --- | --- | --- | --- | --- | --- | --- | --- | --- | --- | --- | --- | --- | --- | --- | --- | --- | --- | --- | --- | --- | --- | --- | --- | --- | --- | --- | --- | --- | --- | --- | --- | --- | --- | --- | --- | --- | --- | --- | --- | --- | --- | --- | --- | --- | --- | --- | --- | --- | --- | --- | --- | --- | --- | --- | --- | --- | --- | --- | --- | --- | --- | --- | --- | --- | --- | --- | --- | --- | --- | --- | --- | --- | --- | --- | --- | --- | --- | --- | --- | --- | --- | --- | --- | --- | --- | --- | --- | --- | --- | --- | --- | --- | --- | --- | --- | --- | --- | --- | --- | --- | --- | --- | --- | --- | --- | --- | --- | --- | --- | --- | --- | --- | --- | --- | --- | --- | --- | --- | --- | --- | --- | --- | --- | --- | --- | --- | --- | --- | --- | --- |
